# Zero-Contrast Left Main Bifurcation PCI in an Elderly Patient with Chronic Kidney Disease

**DOI:** 10.1155/2021/6658992

**Published:** 2021-03-12

**Authors:** Prathap Kumar, Stalin Roy, Blessvin Jino, Manu Rajendran, Sandheep G. Villoth

**Affiliations:** Meditrina Hospital, Kollam, Kerala, India

## Abstract

Patients with chronic kidney disease develop acute kidney injury (AKI) following percutaneous coronary intervention (PCI). We report a case highlighting the benefits of zero-contrast left main bifurcation PCI in an 82-year-old male with non-ST elevation myocardial infarction and contrast-induced AKI following coronary angiography. The patient was on routine follow-up, and he was stable and asymptomatic at nine months follow-up.

## 1. Introduction

Percutaneous coronary intervention (PCI) in patients with significant renal dysfunction poses multiple challenges. The advent of image-guided zero or ultralow contrast angioplasty is beneficial for patients who were previously considered ineligible or at “high risk” for routine coronary angioplasty [1]. Although zero-contrast PCI is a new emerging method in the field of coronary interventions, complex interventions such as PCI for left main coronary artery, difficult chronic total occlusions (CTO), [2], and severely calcified lesion subtypes [3] pose significant challenges when performed without using contrast media/agents. On the other hand, contrast-induced nephropathy (CIN) significantly impacts the long-term prognosis of patients undergoing PCI, and it is independently associated with higher mortality in patients with diabetes [4].

In general, contrast volume should be restricted to twice or thrice the estimated glomerular filtration rate (eGFR). The maximum acceptable contrast dose (MACD) is calculated as 5 ml × body weight (kg)/baseline serum creatinine (mg/dl) [5]. The MACD is validated, and contrast use within the cut-off value reduces the incidence of CIN [6]. Recently, a stringent cut-off MACD value of 3.7 x eGFR has been proposed and endorsed by the Society for Cardiovascular Angiography and Interventions consensus statement [7, 8]. Intravascular ultrasound- (IVUS-) guided PCI significantly reduces the contrast volume compared to the conventional PCI (“Angiography only” approach). Herein, we present a case of zero-contrast left main bifurcation PCI in a patient with non-ST elevation acute coronary syndrome (NSTE ACS) and contrast-induced acute kidney injury (CI-AKI) following coronary angiography (CAG). To the best of our knowledge, this is the first case report of zero-contrast left main bifurcation PCI.

## 2. Case Report

**Figure 1:**
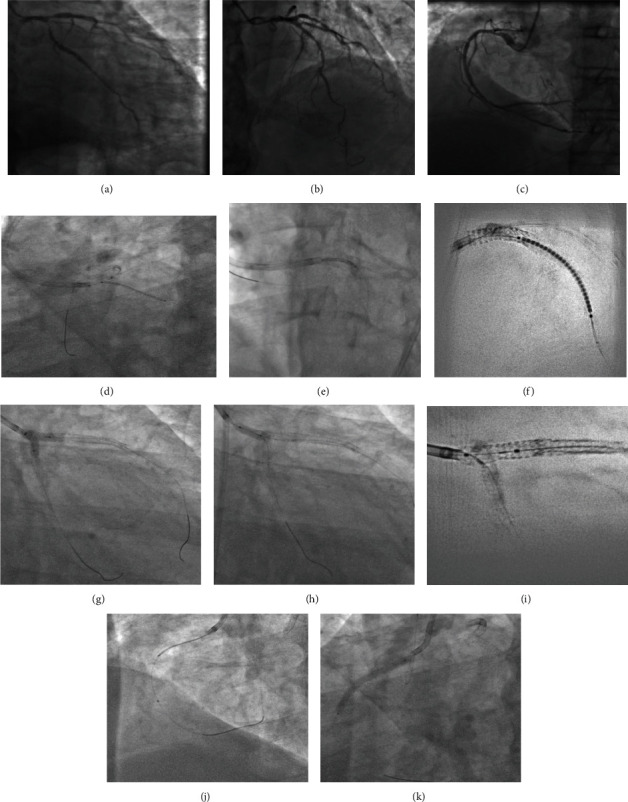
(a) Medina 0,1,1 disease. (b) Significant disease in proximal-mid LAD. (c) RCA proximal-mid disease. (d) Ostial LCX stent. (e) Sepal wire technique for ostial LMCA stent. (f) CLEARstent image of LAD stent. (g) FKBI (h) POT. (i) CLEARstent of bifurcation. (j) IVUS evaluation of RCA disease. (k) Ostial RCA stent.

An 82-year-old male patient with diabetes, hypertension, and chronic kidney disease (CKD) stage III-b (eGFR: 39 ml/min/1.73 m^2^) presented to a primary hospital with NSTE ACS. Coronary angiography revealed critical three-vessel disease involving the ostium of the left anterior descending (LAD), left circumflex (LCX) arteries (medina 0,1,1), and proximal right coronary artery (RCA; Figure 1). However, 48 hours after CAG, serum creatinine levels increased from 178 *μ*mol/l to 290 *μ*mol/l (eGFR, 19 ml/min/1.73 m^2^), indicating CI-AKI. The patient was given the option of coronary artery bypass grafting (CABG)/left main bifurcation PCI and referred to our centre for further management.

**Figure 2:**
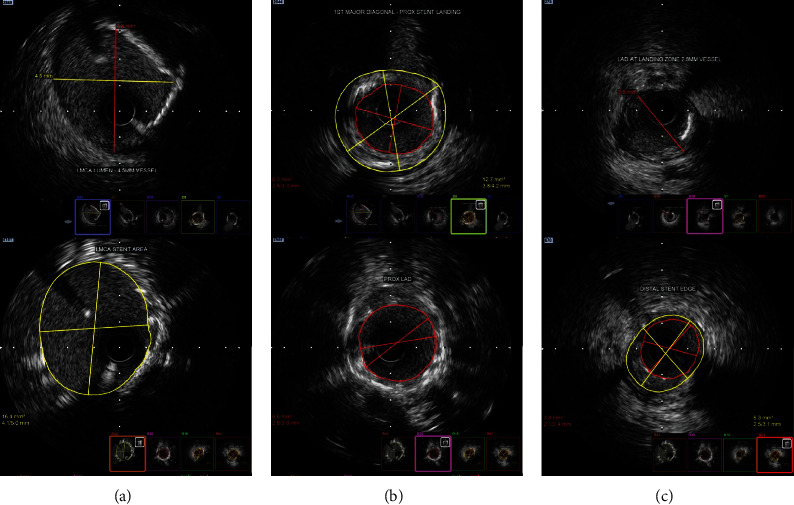
(a) Pre-poststenting images of LMCA. (b) Proximal LAD pre-poststenting. (c) Distal landing zone and stent edge.

**Figure 3:**
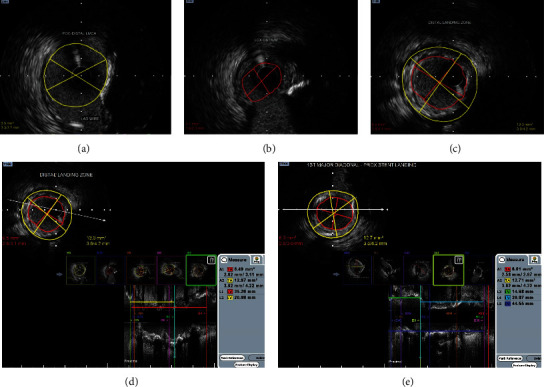
(a) Distal LMCA IVUS. (b) Significant disease at LCX ostium. (c) Distal landing zone in major OM. (d) IVUS image in LCX for assessing stent length. (e) IVUS in LM-LAD to decide proximal and distal stent lengths.

Diagnostic CAG films available from primary centre were reviewed. The patient presented with a SYNTAX score of 25 and a high STS II score (25% morbidity/mortality) and hence was advised to undergo zero-contrast left main bifurcation PCI. The other main factor for choosing PCI over CABG is due to significant past experience in performing ultralow and zero-contrast PCI at our centre. Post consent, the procedure was performed via the femoral approach using a 7Fr 3.5 EBU guide catheter, three days after CAG. Distal landing zones and measurements were obtained from the IVUS runs from LCX and LAD to LMCA (Figures 2 and 3(a)–3(c)). Although LMCA disease was not observed, ostial disease of both LAD and LCX was noted. The LCX and LAD lesions were subsequently predilated with multiple noncompliant (NC) balloons.

Minicrush technique was proposed for the LMCA bifurcation as described in the European Bifurcation Club consensus document [9]. The lesion length in the LCX-OM segment was 21 mm, and the reference luminal diameter at the distal landing zone was 2.9 mm. Hence, a 3 × 24 mm drug-eluting stent (DES) was used for LCX. The stent was placed in the LCX ostium using a balloon in the LMCA-LAD as a marker. The balloon placed in the main vessel (LMCA-LAD) helps us identify the ostium of the side branch (LCX) precisely, so that the LCX stent was deployed at the ostium with minimal protrusion into the LMCA (proximal stent edge positioned just touching the balloon in the LMCA). Then the LCX guidewire was removed. The stent was then crushed using the balloon in the LMCA-LAD. The LMCA-LAD stent chosen was a 4 × 16 mm DES, as the LAD vessel diameter was 4 mm up to the first diagonal branch, and the length from the LMCA ostium to the diagonal was 14.5 mm (Figures 3(d) and 3(e)).

Aorto-ostial stenting is a difficult procedure especially when performed without using contrast media/agents to check the exact stent position while deploying. We used marker wire technique (sepal technique) for optimal positioning of the stent in ostial LMCA and RCA [10]. In this technique of ostial stenting, a second guide wire was placed in the aortic root to mark the ostium of the coronary vessel (LMCA). When the guide catheter is maximally advanced with the secondary guidewire in the aorta, it stops exactly at the ostium of the coronary artery (as further advancement is prevented by the guidewire which is floating in the aorta) (Figure 1(e)). Hence, the tip of the guide catheter serves as the marker for the coronary ostium, from which point the LMCA-LAD stent was deployed; the middistal LAD lesion was reassessed using IVUS for deciding the second stent in LAD. The distal reference diameter was 2.75 mm, and the myocardial bridge was observed distal to the lesion. The length of the lesion from the stent edge was 31 mm. Hence, a 2.75 × 32 mm DES was selected to avoid landing in the myocardial bridge segment, and a precise, minimal overlap was done to ensure entire lesion coverage using the “CLEARstent” technology from Siemens.

The minicrush procedure was completed with proximal optimization technique (POT) followed by recross into the LCX. Subsequently, final kissing balloon inflation (FKBI) was carried out. Final POT was performed using a 4.5 × 8 mm NC balloon followed by IVUS runs of the LAD and LCX arteries. The LMCA ostium was well covered, and there was no malapposition or edge dissection. A few areas of underexpansion were noted in the LAD stent, and the ostial area of LCX was only 4.72 mm^2^. Therefore, postdilatation of these regions was performed again using high-pressure NC balloons. Bifurcation PCI was completed by redoing FKBI, followed by a POT (Figure 1).

Right coronary intervention was subsequently performed; after engaging the RCA, IVUS was done, which showed significant disease starting from the ostioproximal to mid-RCA with a lesion length of 31.5 mm. A 3.5 × 37 mm DES was used. Using the sepal wire technique, the stent was positioned in the RCA ostium and deployed. Postdilatation was performed, and final IVUS showed a well-expanded stent with no edge dissection or malapposition, covering the RCA ostium.

It is difficult to identify the exact stent position and perform aorto-ostial stenting without contrast media. We used the marker wire technique for precise positioning of the LMCA and RCA ostial stents. The procedure was completed at a total fluoroscopy time of 36.9 minutes and a radiation dosage of 4434 milligray (mGy) with 0 ml of contrast medium/agents. The patient was discharged 72 hours later, after creatinine levels reduced from 290 *μ*mol/l to 253 *μ*mol/l. The post-PCI eGFR was 23 m/min/1.73 m^2^. No renal replacement therapy was required before or during the hospital stay. The patient visited the hospital periodically for routine evaluation, and after three months, he was totally asymptomatic. His serum creatinine levels stabilized at around 177 *μ*mol/l without the need for hospitalization or renal replacement therapy.

## 3. Discussion

To the best of our knowledge, this is the first case report describing zero-contrast IVUS-guided left main bifurcation PCI. This case substantiates the role of image and physiology-guided zero-contrast PCI in managing highly complex interventions for critical conditions such as left main bifurcation disease. Experts in this area can help in alleviating angina associated with coronary artery disease in patients having significant renal impairment and are ineligible for undergoing PCI for fear of worsening renal function.

Larger catheters are preferred for zero-contrast PCI, as simultaneous use of multiple guidewires and imaging is required. Catheter engagement was confirmed by passing the guidewire using previous roadmaps and comparing the course of the wire in the coronary vessel. Other techniques for confirming guide catheter engagement are injection of cold saline (10–20 ml) and identification of electrocardiogram changes. The saline injection induces T-wave inversion or increases its amplitude along with ST-segment depression or elevation if catheter is properly inserted [3]. During wiring of coronary vessels, the same projections taken in the diagnostic study are used, and reference images are always kept side by side. This step may be challenging when the diagnostic study is performed at a different centre and images cannot be displayed while performing the procedure. This limitation is overcome using additional monitor screens in the Cath Lab.

Intravascular imaging is an important component of zero-contrast PCI. Expertise in IVUS image interpretation, as well as strict vigilance in anticipating, identifying, and successfully managing complications, is of paramount importance. It is recommended that only highly trained IVUS interpreters attempt such complex procedures [2]. The IVUS catheter can be manually positioned at the proximal and distal reference segments, with dry cine taken and used as landmarks for stent deployment. Other techniques for identifying the stent position are using marker wires in major branches and measuring the lengths of such branching points from the stent-landing zone using “marker wire” or “metallic silhouette” technique [11].

The threshold for using contrast in the event of a suspected complication should be extremely low. For clinical signs such as chest pain, ECG changes, or hypotension, a low-contrast check angiogram should be performed immediately, considering the safety of the patient. Poststenting IVUS should be done and reviewed meticulously to check for complications. It is mandatory to use transthoracic echocardiogram immediately postprocedure to evaluate for the presence of pericardial effusion. Finally, fractional flow reserve (FFR) is a highly beneficial tool for confirming the accuracy of PCI.

## 4. Conclusion

Imaging-guided left main bifurcation PCI without the use of contrast was feasible and safe in a patient presenting with multivessel coronary artery disease and CI-AKI. This technique can be used safely in patients who are at high risk for CI-AKI in centres with expertise in complex PCI with intravascular imaging guidance.

## Additional Points

*Learning Objectives*. Intravascular ultrasound-guided zero-contrast PCI is very difficult to accomplish, especially when the left main coronary artery bifurcation is involved. However, steps such as identification of the exact vessel dimensions, lesion length, and planning stent deployment by marking the landing zone using dry cine angiograms while keeping the IVUS catheter, when done by those with significant expertise in IVUS-guided left main interventions, can immensely benefit CKD patients with significant angina.
